# The Two Pandemics

**DOI:** 10.1017/dmp.2020.275

**Published:** 2020-07-27

**Authors:** Luis Alejandro Boccalatte

**Affiliations:** Head and Neck Surgery, Department of General Surgery, Hospital Italiano de Buenos Aires, Argentina and Faculty, Medical School Instituto Universitario Hospital Italiano, Buenos Aires, Argentina

**Keywords:** communication, disease outbreaks, health communication, pandemics

## Abstract

This article reflects on the importance and the impact of scientific publications in the midst of a global health crisis. It aims to raise awareness about the responsibility of accepting manuscripts in such sensitive times and is intended to motivate the production of high-quality papers through a critical vision.

In late December 2019, several local health facilities reported clusters of patients with severe pneumonia of unknown cause in Wuhan, Hubei Province, China.^1^ On December 31, 2019, the Chinese Centers for Disease Control conducted an investigation detecting severe acute respiratory syndrome coronavirus 2 (SARS-CoV-2) as the etiological agent. The World Health Organization declared the coronavirus disease (COVID-19) pandemic on January 30, 2020.^2^

In this context of global uncertainty, we can consider that there are 2 different sides of the same pandemic: first, the spread of SARS CoV-2; second, the “academic pandemic” resulting from an exponential increase in the number of articles published in international medical journals (Figure 1).

**FIGURE 1:**
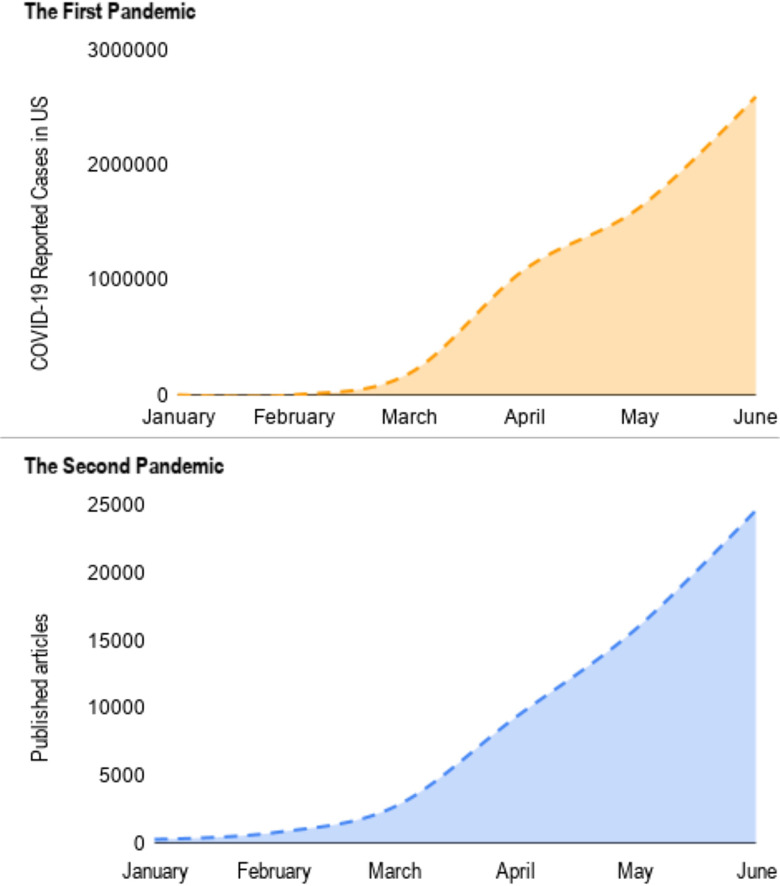
The spread of COVID-19 Reported Cases in US and Exponential Increase in Articles Published in International Medical Journals.

The pandemic per se, the first one, spread around the world reaching, at the moment of this letter, 11 304 534 confirmed cases, 531 659 deaths, and 6 111 195 recovered patients in 188 countries/regions. For this reason, it is essential for the health workforce to be up-to-date and supported by the latest available information to take evidence-based actions. The scientific community understands that, due to the urgent need for data regarding diagnostic methods, risk factors, and treatments, research articles have fast track peer-reviews in order to obtain accurate data for daily practice. The dynamic and uncertain situation causes many manuscripts of dubious methodology to propose unconventional therapies, present unusual associations, and force explanations that are accepted in extremely short periods of time.

This leads to the second pandemic, the academic one, which plunges physicians and scientists with an overwhelming number of reports each day, corrected and accepted by journals in a matter of days. Now, does the increasing amount of publications mean that doctors rely more on the main scientific journals? In the same way that social distancing prevents the spread of the virus, this second pandemic causes practitioners to move away from the unsustainable volume of papers that reach their mailboxes.

From the beginning of the pandemic, the amount of manuscripts published on PubMed (without considering those accepted in unindexed literature and those still undergoing proofreading) have reached 29 034 when sought as “(COVID-19)” AND ([“2019/12/31“(Date – Publication): “3000“(Date – Publication)]) and 14 725 as “(SARS-CoV-2),” resulting in an average of 154.4 publications each day.

The question is: Which pandemic is the most dangerous? Which one is most threatening? The COVID-19 pandemic will surely have an immeasurable impact on society at a global scale, as well as in the health care and education systems,^3^ which is expected in the context of a health emergency. However, it is necessary to ask ourselves: Why do we relegate the quality we have always been working for? What is the cure for this second pandemic? Will we have to modify the way we test authors or journals when it comes to evaluating if they are infected with this second “academic virus”?

It is necessary to revalue the importance of quality in reporting and research, setting aside the concept that a paper is worth publishing for the sole reason that it was written by an experienced researcher or conducted in a prestigious institution, keeping always a critical blinded analysis.^4^

This letter has the humble objective of praising high-quality scientific works, promoting reflection in times of global crisis, being critical of our peers, thinking about medical education and its impact, and bearing in mind that perhaps pandemics have neither 1 nor 2 but several viruses for which we must find the cure.
